# Influence of specific collagen peptides and 12-week concurrent training on recovery-related biomechanical characteristics following exercise-induced muscle damage—A randomized controlled trial

**DOI:** 10.3389/fnut.2023.1266056

**Published:** 2023-11-16

**Authors:** Kevin Bischof, Savvas Stafilidis, Larissa Bundschuh, Steffen Oesser, Arnold Baca, Daniel König

**Affiliations:** ^1^Section for Nutrition, Exercise and Health, Department of Sports Science, Centre for Sports Science and University Sports, University of Vienna, Vienna, Austria; ^2^Vienna Doctoral School of Pharmaceutical, Nutritional and Sport Sciences, University of Vienna, Vienna, Austria; ^3^Department for Biomechanics, Kinesiology and Computer Science in Sport, Centre for Sports Science and University Sports, University of Vienna, Vienna, Austria; ^4^Collagen Research Institute, Kiel, Germany; ^5^Section for Nutrition, Exercise and Health, Department of Nutrition, Faculty of Life Sciences, University of Vienna, Vienna, Austria

**Keywords:** specific collagen peptides, recovery, muscle damage, concurrent training, repeated bout effect

## Abstract

**Introduction:**

It has been shown that short-term ingestion of collagen peptides improves markers related to muscular recovery following exercise-induced muscle damage. The objective of the present study was to investigate whether and to what extent a longer-term specific collagen peptide (SCP) supplementation combined with a training intervention influences recovery markers following eccentric exercise-induced muscle damage.

**Methods:**

Fifty-five predominantly sedentary male participants were assigned to consume either 15 g SCP or placebo (PLA) and engage in a concurrent training (CT) intervention (30 min each of resistance and endurance training, 3x/week) for 12 weeks. Before (T1) and after the intervention (T2), eccentric muscle damage was induced by 150 drop jumps. Measurements of maximum voluntary contraction (MVC), rate of force development (RFD), peak RFD, countermovement jump height (CMJ), and muscle soreness (MS) were determined pre-exercise, immediately after exercise, and 24 and 48 h post-exercise. In addition, body composition, including fat mass (FM), fat-free mass (FFM), body cell mass (BCM) and extracellular mass (ECM) were determined at rest both before and after the 12-week intervention period.

**Results:**

Three-way mixed ANOVA showed significant interaction effects in favor of the SCP group. MVC (*p* = 0.02, *ηp*^2^ = 0.11), RFD (*p* < 0.01, *ηp*^2^ = 0.18), peak RFD (*p* < 0.01, *ηp*^2^ = 0.15), and CMJ height (*p* = 0.046, *ηp*^2^ = 0.06) recovered significantly faster in the SCP group. No effects were found for muscle soreness (*p* = 0.66) and body composition (FM: *p* = 0.41, FFM: *p* = 0.56, BCM: *p* = 0.79, ECM: *p* = 0.58).

**Conclusion:**

In summary, the results show that combining specific collagen peptide supplementation (SCP) and concurrent training (CT) over a 12-week period significantly improved markers reflecting recovery, specifically in maximal, explosive, and reactive strength. It is hypothesized that prolonged intake of collagen peptides may support muscular adaptations by facilitating remodeling of the extracellular matrix. This, in turn, could enhance the generation of explosive force.

**Clinical trial registration:**

ClinicalTrials.gov, identifier ID: NCT05220371.

## 1. Introduction

As an integral component of connective tissue, collagen accounts for approximately 30% of the total protein mass in the human body (1). In contrast to the amino acid composition of myofibrillar protein, collagen peptides (CP) mainly consist of glycine, proline and hydroxyproline. Collagen is found in various tissues such as cartilage, tendons, ligaments and muscles. It not only provides stability and protection, contributing to the structural integrity of these tissues, but also plays a crucial role in enhancing force transmission. There is a constant process of collagen degradation and synthesis to perform these functions, with a turnover rate of about 0.5–2% per day. This process is influenced by incoming signals from the surrounding extracellular matrix (2, 3). In addition, muscles play a vital role in remodeling intramuscular connective tissue (IMCT), an essential component of the extracellular matrix (ECM). A remodeling process that involves binding skeletal muscle-specific α7β1-integrin to high-affinity ligands such as laminin, fibronectin, and collagen. This interaction triggers signal transduction pathways that subsequently activate the appropriate gene expression patterns, leading to the development of ECM plasticity (4). An optimal tailored and distinct IMCT in which collagen types I and III account for ∼75% (5) is essential for proper mechanotransduction and, thus, optimal movement performance.

Several studies have investigated the influence of specific exercise modalities on the protein synthesis of muscle and tendon connective tissue, the latter acting as a force transmitter between myotendinous junctions and bones. Muscles, tendons, and IMCT of the lower limbs were found to have higher gene expression of certain collagenous tissues and mediators (e.g., collagen type I, III, TGFβ-1, lysyl oxidase) after both resistance and endurance training in rodents and humans, suggesting a possible role in regulating muscle adaptation, repair, recovery and restructuring (2, 5–8). Muscle fiber hypertrophy is not always accompanied by equal turnover rates of ECM components, suggesting different growth potential (9). Recently, supplementation with CP was shown to stimulate collagen synthesis (10). In contrast, the intake of other proteins (e.g., essential amino acids, whey) did not significantly increase MCT synthesis rates, even when combined with exercise (11–14).

The ability to generate force decreases markedly after exercise-induced muscle damage, which persists for up to 7 days (15). In addition to physical treatments (10), nutritional supplementation with various protein sources has been linked to a small association between muscle function recovery, muscle soreness, and indirect markers of muscle damage (16). Recently, CP administration has resulted in significant improvements in handgrip strength (17) and isokinetic quadriceps strength (18), producing similar results to whey protein (19).

Both endurance and strength exercises can induce muscle damage, resulting in local cellular and systemic responses (20). Delayed onset muscle soreness (DOMS), a commonly used marker of muscle damage, usually increases from 8 h, peaks at 24–48 h after exercise (21) and is easily detected. Further research is needed to provide a clear picture of the potential benefits and efficacy concerning recovery from high-intensity exercise after CP supplementation. Four studies (including two pilot studies) have shown a non-significant flattening of the creatine kinase (CK) curve (22), a significant increase in countermovement jump height (CMJ) at 24 h (23) and 48 h (24) and MVC at 48 h (25) after a muscle-damaging exercise bout. Apart from the acute changes, a three-week power training intervention with a daily intake of 20 g CP showed an attenuated decline in RFD during maximal isometric contraction and an improved eccentric deceleration impulse during CMJ compared to the placebo group (26).

Based on the aforementioned findings, there is limited data on recovery from both long-term supplementation and training interventions. Therefore, the objective of the present study was to investigate whether daily CP supplementation is superior to placebo in promoting recovery after a 3-month training intervention. To this end, data from performance tests such as MVC, RFD, and CMJ and perceived muscle soreness will be collected that may provide insight into potential myofibrillar and IMCT adaptations that could indicate improved recovery.

## 2. Materials and methods

### 2.1. Experimental design and participants

The study was conducted as a single-center, double-blind, placebo-controlled, randomized, prospective trial at the University of Vienna, Austria. Before recruitment, the study was approved by the Ethics Committee of the University of Vienna (reference no. 00765) and registered at ClinicalTrials.gov (ID: NCT05220371).

A total of 75 healthy male participants aged 18–40 years who had not exercised more than 3 h per week in the preceding weeks, as determined by the IPAQ questionnaire, were randomized after signing a written informal consent. Exclusion criteria for the study included BMI less than 18.5 kg/m^2^ or greater than 25 kg/m^2^, unstable weight and dietary patterns, symptoms related to physical activity, arterial hypertension, diabetes mellitus, and use of protein supplements within the 6 months before the start of the study. Following American College of Sports Medicine guidelines, individuals with cardiovascular, renal, or metabolic diseases were excluded from the study (ACSM) (27).

**FIGURE 1:**
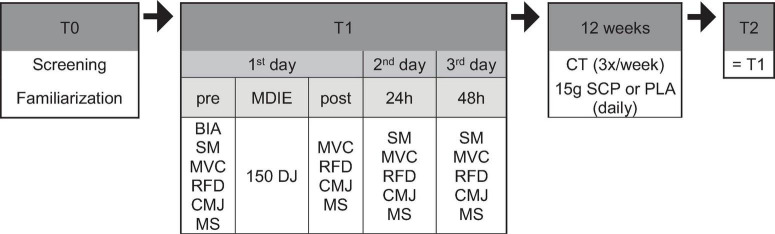
Study design in chronological order. BIA, bioelectrical impedance analysis; MVC, maximum voluntary contraction; RFD, rate of force development; CMJ, countermovement jump; MS, muscle soreness; MDIE, muscle damage-inducing exercise; DJ, drop jump; CT, concurrent training; SM, standardized meal.

Figure 1 depicts the study protocol, which consisted of pre-tests conducted before the training intervention, a 12-week training intervention with a daily SCP or PLA supplementation phase and post-tests conducted after completion of the training intervention. Two weeks before the start of the study (T0), subjects were examined, introduced to the testing procedures, familiarized with the testing equipment, and visited the biomechanics laboratory where all T1 (baseline) and T2 (after 12 weeks) tests were performed. On day 1 of T1, subjects arrived at the laboratory between 7 and 9 am after an overnight fast. A bioelectrical impedance analysis (BIA) was performed to obtain body composition, and a standardized meal (SM) was provided. Participants were required to complete a 5-min warm-up program on a rowing ergometer at moderate intensity one and a half hours after SM. Then, MVC, RFD and CMJ (in this exact order) were performed (“pre”). After the last CMJ, a visual analog scale was used to measure each subject’s perception of muscle soreness (MS). After that, 150 drop jumps were performed. Thirty minutes later, MVC, RFD, CMJ and MS were measured again to get “post” results. On days 2 and 3, participants visited the laboratory at the same time as day 1, received their SM, warmed up for 5 min, and performed the same tests (MVC, RFD, CMJ, and MS) to obtain “24 h” and “48 h” values. During T1 and the last 48 h before day 1, participants were asked to abstain from moderate to vigorous physical activity and alcohol to rule out any influence on recovery. From arrival until leaving the biomechanics laboratory on the three consecutive days, they were only allowed to drink water after consuming the standardized meal. After the 12-week training intervention, the same procedure as in T1 was repeated. A room temperature of 20–22°C was ensured on test days.

### 2.2. Muscle damage-inducing exercise (MDIE)

Part of the pre-test and post-test were 150 drop jumps (6 sets of 25 repetitions, 2 min rest between sets) from a 60 cm high box. Participants were required to land slowly and in a controlled manner at a knee angle of at least 90° to force an adequate eccentric movement and subsequently induce muscle damage, as described elsewhere (28).

### 2.3. Training intervention

The 12-week training was a concurrent training (CT) session that included both strength and endurance elements, in which the SCP and PLA groups participated to the same extent. Before a 30-min run at a specific heart rate, calculated for each participant using the Karvonen formula (29) (exercise intensity factor between 0.7–0.8; HRmax = 220—age) and monitored by a wrist-mounted fitness tracker, subjects were instructed to perform bodyweight-based lower-body exercises (squats, lunges and calf raises) three times a week, with at least 1 day off between two training days. Weeks 1–4 required 3 sets of 20, weeks 5–8 required 3 sets of 25, and weeks 9–12 required 3 sets of 30 repetitions for each exercise. For endurance training, the individual training intensity factor was increased by 5% after every 4th week. The training sessions took place outdoors on Mondays, Wednesdays and Fridays at 7 am and 6 pm under the supervision of sports scientists at the Institute of Sport Science in Vienna and lasted approximately 1 h. Participants were instructed to exercise regularly, either in the morning or afternoon. A minimum of 26 training sessions were required to successfully complete the study.

### 2.4. Dietary intake

Subjects were asked to record their food intake on 2 weekdays and one weekend day in week 1 and 12 to gain insight into their dietary habits. Participants noted every meal and beverage they consumed as well as their weight/volume. Nut.s (nutritional.software, dato Denkwerkzeuge, Vienna) was used to calculate total energy intake, fat, protein and carbohydrate.

### 2.5. Standardized meal (SM)

On each test day, participants consumed an SM consisting of oats and an oat drink 2 h before the first examination. The standardized meal contained 1 g carbohydrate per kg of body weight. At T2, the subjects were also required to take PLA or SCP with their meal for three consecutive days. Participants were prohibited from eating or drinking for 12 h before SM (water excluded on days without BIA measurements). Alcohol consumption was also prohibited 48 h before and during the test days.

### 2.6. Supplementation

The subjects were randomly assigned to the SCP or PLA group using a research randomizer.^1^ Once a day for 12 weeks, they were required to take 15 g of either a placebo (Silicea, metabolically inert) or the test product [mixture of specific collagen peptides (10 g PeptENDURE^®^ + 5 g TENDOFORTE^®^), Gelita AG, Eberbach, Germany] orally, both similar in taste and consistency (powder), in combination with 0.5 l water. On training days, 7.5 g of the supplement or placebo had to be taken 1 h before and 7.5 g immediately after the training session to ensure high blood SCP levels during and after the training (30–32). On non-exercise days, participants were asked to consume 15 g as a whole at the same time as on exercise days. At the end of the study (T2), the supplements were taken in combination with the standardized meal before the examination. The amino acid composition of the SCP is listed in Table 1.

**TABLE 1:** Amino acid composition of the specific collagen peptides.

Amino acid	Weight (%)
Hydroxyproline	11.3
Aspartic acid	5.8
Serine	3.2
Glutamic acid	10.1
Glycine	22.1
Histidine	1.2
Arginine	7.8
Threonine	1.8
Alanine	8.5
Proline	12.3
Tyrosine	0.9
Hydroxylysine	1.7
Valine	2.4
Methionine	0.9
Lysine	3.8
Isoleucine	1.3
Leucine	2.7
Phenylalanine	2.1

### 2.7. Body composition

BIA (Nutriguard MS, data-input GmbH, Germany) with a frequency of 50 kHz was used to analyze body composition. ECM (extracellular mass), ICM (intracellular mass), FM (fat mass) and FFM (fat-free mass) were recorded. BIA measurements took place on day 1 of T1 and T2 and were the participants’ first act after entering the laboratory. After an overnight fast (water was also prohibited), the subjects lay on a mat parallel to the ground. They remained there for 5 min without moving to allow optimal body water distribution. To ensure good conductivity between the skin and electrodes, an electrode spray was applied before attaching two electrodes each on the back of the right hand and the right foot and connecting to the BIA device with cables.

### 2.8. Maximum voluntary contraction (MVC), rate of force development (RFD) and peak RFD

Subjects began the trials seated (110° hip angle) on a commercial dynamometer (HUMAC NORM Model 770; CSMi, Stoughton, MA, USA) with their hands crossed over the chest, a fastened four-point seatbelt for the upper body, a fastened strap for the leg, and one just above the ankle. The dynamometer position and knee angle (110° for maximum momentum, 180° = full extension) were individually set at baseline. The same position was used for repeated measurements. After the command “3, 2, 1, go,” the participants extended their right leg as hard and as far as possible for 3 s for the MVC trials. For the RFD trials, subjects contracted their right leg for a very short time (< 200 ms) with the instruction to generate as much force (i.e., as explosive) as possible as quickly as possible (33). Moreover, the investigators strongly encouraged subjects verbally during the contractions while providing simultaneous visual feedback. As with the CMJ, three valid trials were required for each calculation. The pause between trials was 60 s. The analog signal of joint moment was recorded by the Vicon Nexus A/D card (16 bit) at 1,000 Hz. The resulting data were filtered with a cut-off frequency of 20 Hz based on Winter 2009 (34). A valid MVC trial represents a force held constantly over at least 2 s. The mean of the maximum force (N) from three valid trials was then used for further analysis. When measuring the rate of force development, any countermovement of the tested leg was prohibited and resulted in exclusion from the trial. RFD was calculated as the slope of the force-time curve (Δforce/Δt) over the time interval of 0–50 ms, and force onset was automatically determined when the force signal reached a threshold of ∼1.4% of MVC, which is similar to the method described in Aagaard et al. (35). Automatic detection of force onset has already shown high ICC and lower SEM% (36). In addition, the RFD peak (0–100 ms) was also measured, which is a highly reliable parameter (37).

### 2.9. Countermovement jump (CMJ) and muscle soreness (MS)

After the RFD trials, participants performed three valid CMJs per test point (pre, post, 24 h and 48 h) barefoot. Subjects stood with both feet shoulder-width apart on a single force plate and waited for instructions from study personnel. This was followed by the verbal command “3, 2, 1 and jump,” which initiated a countermovement of the participants characterized by a knee angle of 90°. Hands had to be kept on the hips from the beginning until landing. A one-minute break between each trial ensured adequate recovery. After the last CMJ, participants were asked to give feedback on how they felt the pain in their legs during the last jump using a visual analog scale (VAS) with numbers ranging from 0 mm (no pain) to 100 mm (unbearable pain). By applying the impulse-momentum method J = JGRF—JBW = m*vtake-off leading to yflight = ypeak—ytake-off = (vtake-off^2^)/2 g, the CMJ height can be calculated (38). The mean value of three valid jumps was used for the further calculations.

### 2.10. Statistical analysis

Statistical analyses were conducted using IBM SPSS Statistics 23 (IBM SPSS Statistics for Windows, Armonk, NY, USA: IBM Corp.). A 95% confidence interval was chosen, and all data are presented as mean ± standard deviation. For the pairwise Student’s *t*-test, the normal distribution was checked with the Kolmogorov-Smirnov test to detect changes over time. Before the independent *t*-test, variance homogeneity was also checked with Levene’s test. To determine any 3-way interaction effect, a mixed design ANOVA (generalized linear model) was calculated using group (SCP, PLA) as between-subject factor and test time (pre, post, 24 h, 48 h) and study time (T1, T2) as within-subject factors. In addition, the same procedure was performed with test time as a two-subject instead of a four-subject factor to reduce the model (referred to as the “reduced model”), shown in Supplementary File 1. The partial eta squared (*ηp*^2^) was reported as the effect size (small effect: *ηp*^2^ > 0.01, medium effect: *ηp*^2^ > 0.06, large effect: *ηp*^2^ > 0.14) (39). To meet the requirements, Mauchly’s test of sphericity was also applied. In the event of significance, an ε of < 0.75 led to the Greenhouse-Geisser correction, > 0.75 to that of Huynh Feldt (40). Because the compared groups in the mixed design ANOVA were almost equal in number (largest/smallest < 1.5), indicating robustness of the F-statistics (41), the homogeneity of variance was not checked with Levene’s test. All tests were two-sided. *Post hoc* multiple comparisons to illustrate differences between times, groups and within times were performed with the Fisher’s least significant difference (LSD) test. For a better illustration of each biomechanical parameter (MVC, RFD, peak RFD, CMJ), the graphs were based on the following procedure: “pre” T1 and T2 were set to 100% for each group. “Post,” “24 h” and “48 h” of T1 and T2 were then normalized for “pre” T1 or T2, resulting in a positive or negative% value. The difference (Δ) of each time point was then calculated (e.g., % of post T2 minus% post T1), resulting in an absolute percentage value (=“normalized delta”). The mean of all cases per time point and group is shown in Figures 3–6. *Post hoc* power analysis was calculated using GPower 3.1.9.7 (Franz Faul, University of Kiel, Germany) and CMJ as parameters. Additional statistical tests of study time × group, study time × test time, group × test time, main effects and numbers without normalized deltas can be found in Supplementary File 1.

**FIGURE 2:**
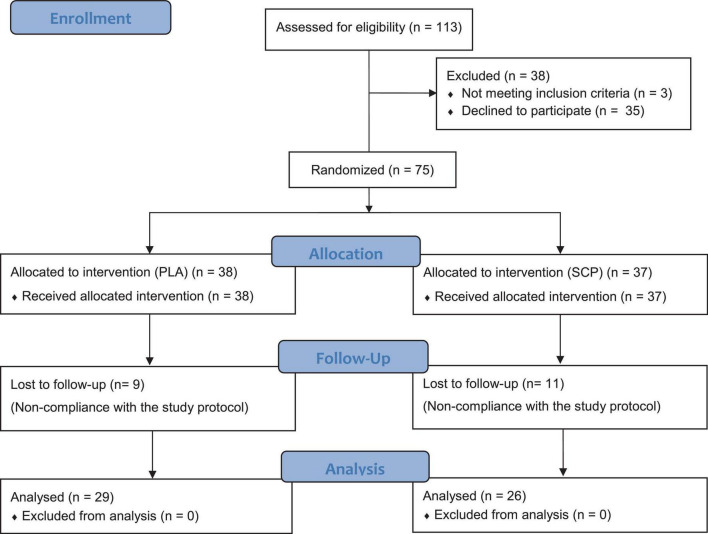
CONSORT flow chart.

**FIGURE 3:**
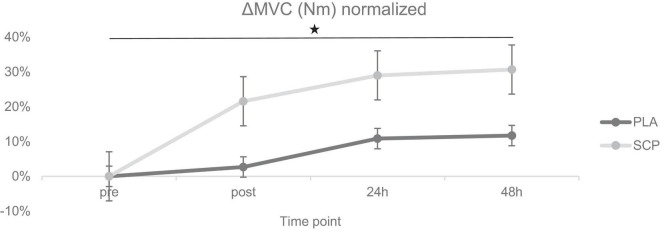
ΔMVC (Nm) normalized + standard errors (SE) for each time point demonstrating a significant 3-way interaction effect (*p = 0.02).

**FIGURE 4:**
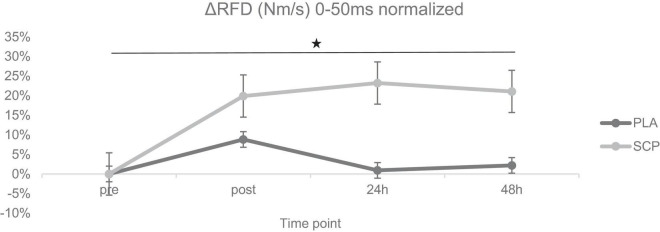
ΔRFD (Nm/s) 0–50 ms normalized + SE for each time point demonstrating a significant 3-way interaction effect (*p < 0.01).

**FIGURE 5:**
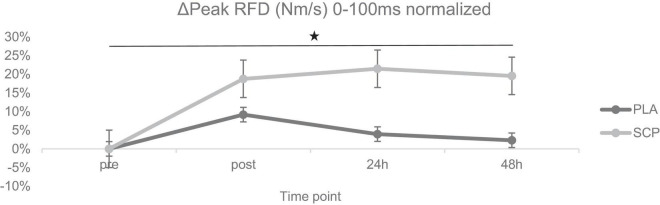
ΔPeak RFD (Nm/s) 0–100 ms normalized + SE for each time point demonstrating a significant 3-way interaction effect (*p < 0.01).

**FIGURE 6:**
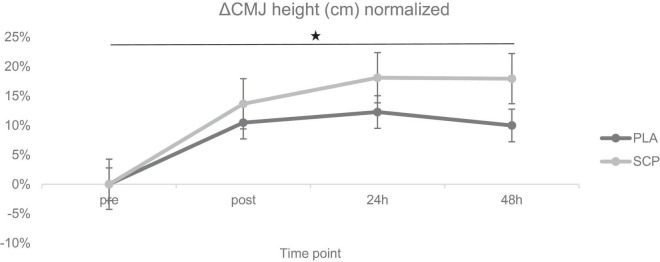
ΔCMJ height (cm) normalized + SE for each time point demonstrating a significant 3-way interaction effect (*p = 0.046).

## 3. Results

After eligibility testing and positive medical examination, a total of 75 subjects were enrolled, randomized, and allocated to either the SCP (*n* = 37) or PLA group (*n* = 38) (Figure 2). One participant failed to show up shortly before randomization, so an odd number of subjects were included. Participants lost to follow-up were eliminated for missing too many training sessions (> 10 times). No adverse events or side effects related to SCP or PLA intake were recorded. The per-protocol analysis consisted of 55 participants in total. Participants in the SCP and PLA groups exercised 32.88 and 33.03 times, respectively, during the 12-week intervention (presented as mean). The independent *t*-test revealed no significant difference (*p* = 0.844) between groups.

### 3.1. Participant characteristics

Regarding baseline anthropometric data, there were no significant differences in age (*p* = 0.44), mass (*p* = 0.35), height (*p* = 0.51), BMI (*p* = 0.7), FM (*p* = 0.27) and FFM (*p* = 0.53) (Table 2).

**TABLE 2:** Participant characteristics represented as mean ± SD.

Variable	Mean ± SD		Group effect (*p*-value)
	SCP (*n* = 26)	PLA (*n* = 29)	
Age (years)	26.1 ± 5.1	27.2 ± 5.2	0.44
Body mass (kg)	75.0 ± 10.0	77.1 ± 6.3	0.35
Height (m)	1.81 ± 0.1	1.82 ± 0.1	0.51
BMI (kg/m2)	22.8 ± 2	23.3 ± 1.8	0.7
FM (kg)	15.2 ± 5.4	16.3 ± 4	0.27
FFM (kg)	59.8 ± 7.3	61.1 ± 4	0.53

SCP, specific collagen peptides; PLA, placebo; BMI, body mass index; FM, fat mass; FFM, fat-free mass.

### 3.2. Body composition analysis

One participant in the PLA group was excluded due to an error in data recording. There was no significant time, group or interaction effect for the body composition variables. SCP and PLA did not significantly differ (Table 3).

**TABLE 3:** Body composition analysis represented as mean ± SD.

Variable	SCP (*n* = 26)		PLA (*n* = 28)		Interaction effect (*p*-value)
	T1	T2	T1	T2	
FM (kg)	15.2 ± 5.4	14.2 ± 5.1	16.5 ± 4	15.8 ± 4.1	0.41
FFM (kg)	59.8 ± 7.3	60.7 ± 7.5	60.7 ± 4.3	61.4 ± 4.2	0.56
ECM (kg)	26.8 ± 3.4	27 ± 3.7	27.7 ± 3	27.7 ± 2.6	0.58
BCM (kg)	33 ± 2.4	33.7 ± 4.7	33 ± 2.4	33.7 ± 2.6	0.79

SCP, specific collagen peptides; PLA, placebo; FM, fat mass; FFM, fat-free mass; ECM, extracellular mass; BCM, body cell mass.

### 3.3. Dietary intake

As illustrated in Table 4, no significant time, group or interaction effect was calculated for either the PLA or the SCP group concerning absolute and relative (to body weight) dietary intake.

**TABLE 4:** Absolute and relative (rel.) dietary intake without supplement represented as mean ± SD.

Variable	SCP (*n* = 26)		PLA (*n* = 29)		Interaction effect (*p*-value)
	T1	T2	T1	T2	
Energy (kcal)	2,276 ± 663	2,104 ± 429	2,360 ± 640	2,404 ± 513	0.24
Protein (g)	91 ± 31	85 ± 20	93 ± 33	98 ± 23	0.32
Fat (g)	83 ± 25	81 ± 20	89 ± 38	96 ± 30	0.36
Carbohydrate (g)	261 ± 91	234 ± 77	265 ± 58	250 ± 52	0.59
Energy rel. (kcal/kg)	31 ± 10	28.6 ± 6.8	30.2 ± 8.4	31 ± 7.2	0.19
Protein rel. (g/kg)	1.23 ± 0.5	1.15 ± 0.3	1.18 ± 0.4	1.26 ± 0.3	0.26
Fat rel. (g/kg)	1.14 ± 0.4	1.11 ± 0.4	1.14 ± 0.5	1.24 ± 0.4	0.30
Carbohydrate rel. (g/kg)	3.53 ± 1.2	3.17 ± 1	3.4 ± 0.8	3.22 ± 0.8	0.52

SCP, specific collagen peptides; PLA, placebo. Units of relative (rel.) variables are related to kg of body weight.

### 3.4. Maximum voluntary contraction (MVC)

Due to technical issues on two test days, data were either recorded erroneously or not recorded at all. Therefore, data from 40 participants were used for analysis of MVC and RFD. Mixed ANOVA showed a statistically significant 3-way interaction effect between study time (T1/T2), test time (pre/post/24 h/48 h), and group (SCP/PLA): Greenhouse–Geisser F(1.659, 63.032) = 4.526, *p* = 0.02, *ηp*^2^ = 0.11. Normalized deltas were also calculated for each time point for better illustration (Figure 3). Regarding study time (T1 vs. T2) in PLA, a significant difference was found for 24 h (*p* = 0.01) and 48 h (*p* < 0.01), while in MVC, there was no effect in pre (*p* = 0.14) and post (*p* = 0.20) (Table 5). SCP showed significant differences post (*p* < 0.01), 24 h (*p* < 0.01) and 48 h (*p* < 0.01) but not pre (*p* = 0.94). For each time point within the study period (T1 and T2), no differences were found between groups [T1 pre (*p* = 0.58), post (*p* = 0.41), 24 h (*p* = 0.12), 48 h (*p* = 0.12), T2 pre (*p* = 0.63), post (*p* = 0.86), 24 h (*p* = 0.72), 48 h (*p* = 0.57)]. In addition, a comparison of pre with post (*p* < 0.01) and 24 h (*p* = 0.01) showed a significant difference in PLA T1 but not in 48 h (*p* = 0.06). In T2, only post (*p* < 0.01) but not 24 h (*p* = 0.25) and 48 h (*p* = 0.69) values were significantly different from pre. In SCP T1, post (*p* < 0.01), 24 h (*p* < 0.01) and 48 h (*p* < 0.01) showed significant differences compared to pre, while in T2, only post (*p* < 0.01) but not 24 h (*p* = 0.22) and 48 h (*p* = 0.68) was significant.

**TABLE 5:** MVC (Nm) represented as mean ± SD.

Group	MVC (Nm)								Interaction effect
	T1				T2				3-way
	Pre	Post	24 h	48 h	Pre	Post	24 h	48 h	
SCP (*n* = 19)	235 ± 52	170 ± 58a	162 ± 54a	170 ± 59a	236 ± 61	217 ± 59b	230 ± 60*	238 ± 54*	*p* = 0.02° (*ηp*^2^ = 0.11)
PLA (*n* = 21)	225 ± 57	186 ± 59a	193 ± 66a	203 ± 73	246 ± 70	213 ± 73*b	237 ± 61*	249 ± 67*	

SCP, specific collagen peptides; PLA, placebo; °, significant 3-way interaction effect. Post-hoc tests: *, significant within groups effect (T1 vs. T2); a, significant within groups effect (T1: pre vs. post, 24 h, 48 h); b, significant within groups effect (T2: pre vs. post, 24 h, 48 h).

### 3.5. Rate of force development (RFD)

Mixed ANOVA revealed a statistically significant 3-way interaction effect between study time (T1/T2), test time (pre/post/24 h/48 h) and group (SCP/PLA): F(3, 114) = 8.398, *p* < 0.01, *ηp*^2^ = 0.18. Normalized deltas for better visualization were also calculated for each time point (Figure 4). Significant differences were also found in RFD in SCP [T1 vs. T2; post (*p* < 0.01), 24 h (*p* < 0.01), 48 h (*p* < 0.01)], but none in SCP pre (*p* = 0.13) and PLA in pre (*p* = 0.13), post (*p* = 0.08), 24 h (*p* = 0.62) and 48 h (*p* = 0.63) (Table 6). Within the study period, significant differences occurred in PLA T1 pre vs. post (*p* < 0.01), 24 h (*p* = 0.05) but not in 48 h (*p* = 0.16); T2 pre vs. post (*p* = 0.01) but not in 24 h (*p* = 0.09) and 48 h (*p* = 0.36). In SCP T1, pre vs. post (*p* < 0.01), 24 h (*p* < 0.01) and 48 h (*p* < 0.01) as well as in T2 pre vs. post (*p* = 0.04) were significantly different but not in 24 h (*p* = 0.87) and 48 h (*p* = 0.64) T2. There were no differences between groups [SCP vs. PLA T1 pre (*p* = 0.37), post (*p* = 0.26), 24 h (*p* = 0.06), 48 h (*p* = 0.12); T2 pre (*p* = 0.62), post (*p* = 0.53), 24 h (*p* = 0.29), 48 h (*p* = 0.67)].

**TABLE 6:** RFD (Nm/s) 0–50 ms represented as mean ± SD.

Group	RFD (Nm/s) 0–50 ms								Interaction effect
	T1				T2				3-way
	Pre	Post	24 h	48 h	Pre	Post	24 h	48 h	
SCP (*n* = 19)	1,994 ± 446	1,469 ± 348a	1,513 ± 416a	1,591 ± 388a	1,872 ± 398	1,755 ± 345*b	1,861 ± 459*	1,901 ± 395*	*p* < 0.01° (*ηp*^2^ = 0.18)
PLA (*n* = 21)	1,883 ± 296	1,593 ± 328a	1,764 ± 388a	1,786 ± 396	1,817 ± 288	1,688 ± 318b	1,720 ± 362	1,749 ± 352	

SCP, specific collagen peptides; PLA, placebo; °, significant 3-way interaction effect. Post-hoc tests: *, significant within groups effect (T1 vs. T2); a, significant within groups effect (T1: pre vs. post, 24 h, 48 h); b, significant within groups effect (T2: pre vs. post, 24 h, 48 h).

### 3.6. Peak RFD

A statistically significant 3-way interaction effect was identified between study time (T1/T2), test time (pre/post/24 h/48 h) and group (SCP/PLA): F(3, 114) = 6.609, *p* < 0.01, *ηp*^2^ = 0.15. Normalized deltas were also calculated for each time point for better visualization (Figure 5). No statistically significant difference was found between groups at T1 pre [(*p* = 0.29), post (*p* = 0.27), 24 h (*p* = 0.12), 48 h (*p* = 0.16)], and T2 [pre (*p* = 0.51), post (*p* = 0.63), 24 h (*p* = 0.34), 48 h (*p* = 0.22)] in peak RFD (Nm/s) 0–100 ms (Table 7). There were differences between groups and study time (T1 vs. T2) in PLA pre (*p* = 0.1) and post (*p* = 0.04) but not in 24 h (*p* = 0.87), 48 h (*p* = 0.7). In SCP, post (*p* < 0.01), 24 h (*p* < 0.01), 48 h (*p* < 0.01) but not pre (*p* = 0.09) was significant. There were differences within the group in PLA T1 pre vs. post (*p* < 0.01), 24 h (*p* = 0.01) but not vs. 48 h (*p* = 0.15). In T2, pre vs. post (*p* = 0.01), 24 h (*p* = 0.13) but not vs. 48 h (*p* = 0.4). In SCP T1 pre vs. post (*p* < 0.01), 24 h (*p* < 0.01), 48 h (*p* < 0.01) and T2 pre vs. post (*p* = 0.01) was significantly different, but not in T2 pre vs. 24 h (*p* = 0.73) and 48 h (*p* = 0.8).

**TABLE 7:** Peak RFD (Nm/s) 0–100 ms represented as mean ± SD.

Group	peak RFD (Nm/s) 0–100 ms								Interaction effect
	T1				T2				3-way
	Pre	Post	24 h	48 h	Pre	Post	24 h	48 h	
SCP (*n* = 19)	2,058 ± 422	1,520 ± 336a	1,587 ± 370a	1,667 ± 365a	1,934 ± 402	1,794 ± 363*b	1,912 ± 452*	1,948 ± 379*	*p* < 0.01° (*ηp*^2^ = 0.15)
PLA (*n* = 21)	1,934 ± 297	1,637 ± 332a	1,776 ± 382a	1,834 ± 381	1,862 ± 272*	1,744 ± 296*b	1,789 ± 342	1,808 ± 330	

SCP, specific collagen peptides; PLA, placebo; °, significant 3-way interaction effect. Post-hoc tests: *, significant within groups effect (T1 vs. T2); a, significant within groups effect (T1: pre vs. post, 24 h, 48 h); b, significant within groups effect (T2: pre vs. post, 24 h, 48 h).

### 3.7. Countermovement jump (CMJ)

Due to illness on two consecutive days, one participant was excluded from the analysis (including the muscle soreness analysis). *Post hoc* power analysis showed a power of 0.959. Mixed ANOVA revealed a statistically significant 3-way interaction effect between study time (T1/T2), test time (pre/post/24 h/48 h) and group (SCP/PLA): Greenhouse–Geisser F(3, 156) = 3.229, *p* = 0.046, *ηp*^2^ = 0.06. Normalized deltas were also calculated for each time point for better illustration (Figure 6). CMJ height differed significantly within groups in PLA T1 pre vs. post (*p* < 0.01), 24 h (*p* < 0.01), 48 h (*p* = 0.02) but not in T2 [pre vs. post (*p* = 0.5), 24 h (*p* = 0.41), 48 h (*p* = 0.15)] (Table 8). In SCP T1 pre vs. post (*p* < 0.01), 24 h (*p* < 0.01) and 48 h (*p* < 0.01) were significantly different, but none in T2 [pre vs. post (*p* = 0.18), 24 h (*p* = 0.74), 48 h (*p* = 0.09)]. Differences between groups and study time (T1 vs. T2) occurred in PLA pre (*p* = 0.04), post (*p* < 0.01), 24 h (*p* < 0.01) and 48 h (*p* < 0.01), in SCP in post (*p* < 0.01), 24 h (*p* < 0.01), 48 h (*p* < 0.01) but not in pre (*p* = 0.09). There were no differences between groups in T1 [pre (*p* = 0.14), post (*p* = 0.97), 24 h (*p* = 0.9), 48 h (*p* = 0.91)] or T2 [pre (*p* = 0.15), post (*p* = 0.37), 24 h (*p* = 0.21), 48 h (*p* = 0.12)].

**TABLE 8:** Countermovement jump height (cm) represented as mean ± SD.

Group	CMJ height (cm)								Interaction effect
	T1				T2				3-way
	Pre	Post	24 h	48 h	Pre	Post	24 h	48 h	
SCP (*n* = 26)	29.7 ± 6.4	25 ± 6.7a	24.2 ± 7.5a	25 ± 6.6a	30.5 ± 6.1	30 ± 6.1*	30.7 ± 6.7*	31.3 ± 5.8*	*p* = 0.046° (*ηp*^2^ = 0.06)
PLA (*n* = 28)	27.2 ± 5.7	24.7 ± 6.9a	24.5 ± 7.5a	25.3 ± 7.5a	28.2 ± 6*	28.4 ± 6.4*	28.5 ± 6.1*	28.8 ± 6.2*	

SCP, specific collagen peptides, PLA, placebo; °, significant 3-way interaction effect. Post-hoc tests: *, significant within groups effect (T1 vs. T2); a, significant within groups effect (T1: pre vs. post, 24 h, 48 h).

### 3.8. Muscle soreness

No significant 3-way interaction effect occurred between study time (T1/T2), test time (pre/post/24 h/48 h) and group (SCP/PLA): F(3, 156) = 0.533, *p* = 0.66, *ηp*^2^ = 0.01. Significant differences between groups in muscle soreness were found in T2 pre (*p* = 0.04) but not in T1 pre (*p* = 0.75), post (*p* = 0.31), 24 h (*p* = 0.95), 48 h (*p* = 0.35), T2 post (*p* = 0.33), 24 h (*p* = 0.48) and 48 h (*p* = 0.54) (Table 9). Within-group differences occurred in PLA T1 pre vs. post (*p* < 0.01), 24 h (*p* < 0.01), 48 h (*p* < 0.01); T2 pre vs. post (*p* < 0.01), 24 h (*p* < 0.01), 48 h (*p* < 0.01); SCP T1 pre vs. post (*p* < 0.01), 24 h (*p* < 0.01), 48 h (*p* < 0.01) and T2 pre vs. post (*p* = 0.01), 24 h (*p* < 0.01), 48 h (*p* < 0.01). Within-group-between-study time (T1 vs. T2) was significantly different in PLA pre (*p* = 0.02), post (*p* = 0.01), 24 h (*p* < 0.01), 48 h (*p* < 0.01); in SCP post (*p* = 0.01), 24 h (*p* < 0.01), 48 h (*p* < 0.01) but not in pre (*p* = 0.65).

**TABLE 9:** Muscle soreness (mm) represented as mean ± SD.

Group	Muscle soreness (mm)								Interaction effect
	T1				T2				3-way
	Pre	Post	24 h	48 h	Pre	Post	24 h	48 h	
SCP (*n* = 26)	0.2 ± 0.3	2.2 ± 1.8a	3.6 ± 2.1a	3.5 ± 1.8a	0.2 ± 0.4^	1 ± 1*ab	1.3 ± 1*ab	1.1 ± 1.2*ab	*p* = 0.66
PLA (*n* = 28)	0.4 ± 0.9	1.8 ± 1.9a	3.6 ± 2.5a	4.1 ± 2.7a	0.1 ± 0.4^*	0.8 ± 0.9*ab	1.1 ± 1*ab	1.2 ± 1*ab	

SCP, specific collagen peptides; PLA, placebo; °, significant 3-way interaction effect. Post-hoc tests: *, significant within groups effect (T1 vs. T2); a, significant within groups effect (T1: pre vs. post, 24 h, 48 h); b, significant within groups effect (T2: pre vs. post, 24 h, 48 h).

## 4. Discussion

The main findings of the study indicate a higher muscular regenerative capacity in subjects supplementing specific collagen peptides (SCP) compared to PLA. This is reflected in significant improvements in the biomechanical parameters maximum voluntary contraction (MVC) and rate of force development (RFD), which recovered significantly faster at each measurement time point (post, 24 and 48 h), and countermovement jump height (CMJ) at 48 h following 150 drop jumps in the SCP group. To the authors’ knowledge, this is the first study to examine the effects of daily SCP supplementation combined with a 12-week training intervention on parameters associated with muscular recovery.

Previous studies have provided evidence of the acute effects of CP intake and shown an increase in CMJ height after 1 week of supplementation. Prowting et al. (23) and Clifford et al. (24) demonstrated a significant interaction effect (time*group) in favor of CP 24 and 48 h, respectively, after a muscle-damaging exercise bout of 100–150 drop jumps. They concluded that ECM remodeling occurred more rapidly in the passive/elastic tissues in the CP group, resulting in improved force output and power production. Another short-term crossover study found that eight intense training sessions before post-test sessions resulted in a similar CMJ height after 24 h and a more pronounced but non-significant recovery after 48 h in the CP group compared to whey protein supplementation (19). In addition to these acute effects, our results suggest a long-term improvement in reactive strength (CMJ height) following SCP administration one and 2 days after muscle-damaging exercise. Higher stiffness results in more efficient use of elastic energy but is reduced during fatigue states (42). Therefore, it appears that chronic supplementation of SCP resulted in increased stiffness of the force-reinforcing collagenous structures of the lower limbs, reducing their sensitivity to the effects of 150 drop jumps. Dressler et al. (43) demonstrated such adaptations after 6 months of CP supplementation in an athlete population, which led to a slight—although non-significant–increase in ankle stiffness compared to a placebo control.

Maximal strength has been shown to be moderately related to reactive strength (44). In the present study, improvements in MVC recovery were significantly higher in the SCP group than in the placebo group. Despite the lack of statistical significance, the investigation by Oertzen-Hagemann et al. (45) in a group of recreationally active men showed that the additional intake of 15 g CP resulted in greater increases in muscle strength than hypertrophy resistance training (RT) alone. In contrast, previous findings suggest that short-term CP supplementation does not further enhance MVC after muscle-damaging eccentric movements (23, 24) or intense RT (19). Jacinto et al. (46) showed a similar but non-significant behavior of MVC after supplementation (35 g/day) of CP compared to whey protein after 10 weeks of RT. However, the change in muscle thickness, which contributes significantly to maximal strength, was significantly higher in the whey protein group than in the CP group. Since there were no obvious changes in BCM, which is mainly composed of muscle tissue, in both groups, the present study design might lead to a better preservation of neural drive in the CP group.

Neural adaptations, especially motor neuron recruitment speed and discharge rate, strongly affect explosive strength, which is usually determined by measuring RFD in a much shorter time interval (< 200 ms) than MVC (37, 47). The shorter the interval, the smaller the deviation from MVC and thus from muscle size (48) which is why a window of 0–50 ms and 0–100 ms (for peak RFD) was chosen to determine whether SCP affects neural-dependent mechanisms (49). SCP supplementation had a pronounced positive effect on RFD, corroborated by a significant 3-way interaction effect. This is in accordance with Lis et al. (26), who demonstrated a significantly higher percent change in RFD (100 ms) in the CP group compared to PLA after 3 weeks of power training and daily ingestion of 20 g CP. The 50 ms RFD time interval was recently shown to correlate strongly with early (0–50 ms) EMG activity at different knee angles (30°, 45°, 60°, 75°, and 90°), suggesting, in particular, a major contribution of muscle unit discharge rate to early RFD (50). Although there is limited evidence, muscle fiber types may also influence the magnitude of explosive contractions. Type II muscle fibers exhibit a higher rate of tension development and cross-bridge cycling (33). Nonetheless, the high variability in fiber type composition between subjects appears to be problematic in defining factors that contribute to RFD (51). Therefore, it remains unclear whether SCP also influences fiber type-specific neural responses. Muscle-tendon stiffness may also play a role, as force transmission is enhanced primarily in stiffer junctions. Bojsen-Møller et al. (52) investigated the mechanical properties of the vastus lateralis tendon-aponeurosis complex and its effects on biomechanical parameters. They found a significant correlation between stiffness and RFD_100 ms_, which describes 30% of the variance in RFD in a sample size of 15 subjects. However, caution should be exercised when attributing stiffness to RFD because inter-individual variability is higher than that of trainability (33).

In an athlete population with exercise-related knee pain, CP has been used successfully to significantly reduce pain during activity after 12 weeks and 5 g of CP daily (53, 54). In the present study, participants reported their perceived muscle soreness after all tests were performed at each time point. Activity-related pain would have impacted the outcome of CMJ, a high range-of-motion exercise. However, the muscle soreness data did not differ significantly, suggesting that only subjective pain was recorded, which SCP supplementation may not affect.

Regarding body composition adaptation, a 12-week RT intervention combined with 15 g of CP has already resulted in a significant increase of FFM and a decrease in FM in premenopausal women, elderly, sarcopenic, and untrained middle-aged men (17, 18, 53, 54) (respectively). In contrast, no significant changes in body composition were observed in the SCP and PLA groups in the present study. This may be because CT produced less myofibrillar hypertrophy than RT alone due to the lower volume of RT-based stimuli, which could explain the non-significant difference. In addition, a higher repetition range (20–30, depending on study time) per set and exercise was prescribed, rather than a moderate repetition range of 6–12, recommended for optimizing muscle hypertrophy (55). The present study was primarily concerned with muscle recovery rather than hypertrophy. Therefore, CT rather than RT was used as the training approach. Apart from probably insufficient hypertrophic stimuli, the daily ingestion of 15 g SCP, which is equivalent to ∼ 60 kcal according to the Atwater factors (56), also caused no significant changes in body composition. Only a slightly more pronounced (absolute) increase in FFM/decrease in FM was observed in the SCP group (Table 2). A 12-week CT combined with 15 g of SCP already significantly increased FFM compared to a non-caloric placebo, at least in experienced recreational female runners (57). In contrast to the present study, the women performed a one-hour endurance run three times a week after RT. The authors mentioned a possible beneficial effect of SCP on peroxisome proliferator co-activator-1a (PGC-1a) and peroxisome proliferator-activated receptor (PPAR), both of which are involved in fat metabolism and stimulation of fatty acid utilization, which has also been demonstrated in mice (58). Perhaps the duration of CT in the current study was too short to induce body compositional adaptations in combination with SCP.

The choice of CT as a training intervention is associated with several physiological adaptations and benefits, most of which are a combination rather than a compromise of those typically resulting from RT or endurance training (ET) alone. A recent systematic review and meta-analysis indicated similar maximal strength and muscle hypertrophy development compared with RT alone (59). Another review confirmed major effects on muscle strength after > 12-week CTs, 30–60 min duration, three sessions per week with high ET intensity and strength before ET (60). However, CT has a negative effect on explosive strength (59), so in the present study, intra-session RT was performed before ET, which could mitigate these impairments. It has been shown that at least 1-RM performance increased significantly when RT was performed in CT before ET (61). Taking this into account, the magnitude of difference over time in RFD in the SCP group is remarkable and supports the possible effect of SCP on explosive strength.

Recovery from exercise-induced muscle damage represents a comprehensive process that occurs in multiple phases and is therefore difficult to assess in its entirety. To date, satellite cells, inflammatory cells, fibro-adipogenic-precursors, vascular tissue, nerve-muscle interaction and the ECM appear to play a central role, not only in the recovery of functionality but also in tissue adaptation to new mechanical stimuli (62). The repeated bout effect (RBE), as a recovery-specific concept and multi-causal physiological mechanism that is not yet fully understood, provides enhanced protection after unaccustomed eccentric exercise, as evidenced by reduced performance decrements, particularly in biomechanical parameters after the second bout. Neural and musculotendinous adaptations, remodeling of the ECM, and inflammation likely regulate RBE (63). Some factors have already been influenced by CP supplementation and therefore represent a promising approach to improving RBE. Since the recorded parameters (CMJ, MVC, RFD) that resulted in significant effects in the present study are biomechanical, improved neural, ECM and/or musculotendinous, complex adaptations are plausible. In the context of ECM remodeling after heavy mechanical loading, the musculotendinous junction (MTJ) displacement recorded in B-mode ultrasound was barely detectable in the biceps brachii 4 weeks after a second bout. The authors suspected that more compliant tendons and muscles were responsible for the apparent change in MTJ displacement (64). As little as 5 g of SCP instead of PLA for 14 weeks significantly increased Achilles tendon cross-sectional area (CSA) but not stiffness and gastrocnemius muscle thickness, once again indicating a potential benefit of SCP in relation to RBE (65). RFD indirectly determined neural adaptations that contributed to the improvement of RBE in the present study and showed a large interaction effect in favor of SCP. No study to date has investigated the effects of CP on neural activation. Therefore, an influence on changes in the activation pattern of motor units as a kind of compensatory strategy, faster recovery of excitation-contraction coupling and/or increased excitability of spinal and motor neurons seem plausible (63, 66, 67). Overall, long-term intake of SCP leads to a significant increase in RBE and thus to improved recovery after exercise-induced muscle damage.

### 4.1. Limitations

The dropout rate of 28% is higher than in other studies with a 12-week training intervention, which limits the statistical power. Some participants contracted COVID-19 during the study, and participants who had just recovered from a mild infection may not have been able to fully adhere to the study protocol in the following week. This, and the fact that they opted for CT instead of RT, may have led to less adaptation of body composition. The determination of subjective muscle soreness using VAS appeared to be error-prone, with some participants entering values above zero before the muscle-damaging bout. Moreover, only male participants were included in the study. The results are not generalizable or applicable to females. Therefore, future studies are strongly recommended to include women (68). The results of the present study are also not fully generalizable to other study results using different CP. Finally, meals were not recorded during the test days (“pre” to “48 h”) at T1 and T2, which may have influenced the performance outcome.

## 5. Conclusion

The findings of this study provide evidence for the significant improvement in recovery-related parameters after 12 weeks of supplementation with specific collagen peptides (SCP) in combination with concurrent training, particularly in maximal, explosive, and reactive strength. Interestingly, despite the lack of significant differences in fat-free mass, the improvements in regenerative capacity suggest that adaptations in musculotendinous structures and potential neural adaptations may play a key role in the observed results.

In order to attain a more profound understanding of the underlying mechanisms, future studies should explore the specific effects of SCP on neural activity patterns and molecular adaptations that contribute to enhancing force transmission. Such research will contribute to elucidating the precise molecular pathways and neural mechanisms underlying the observed improvements in strength and recovery.

## Data availability statement

The raw data supporting the conclusions of this article will be made available by the authors, without undue reservation.

## Ethics statement

The studies involving humans were approved by the Ethics Committee of the University of Vienna. The studies were conducted in accordance with the local legislation and institutional requirements. The participants provided their written informed consent to participate in this study.

## Author contributions

KB: Conceptualization, Formal analysis, Investigation, Project administration, Visualization, Writing – original draft. SS: Formal analysis, Methodology, Supervision, Validation, Visualization, Writing – review and editing. LB: Investigation, Project administration, Writing – review and editing. SO: Formal analysis, Writing – review and editing. AB: Formal analysis, Writing – review and editing. DK: Conceptualization, Formal analysis, Funding acquisition, Methodology, Resources, Supervision, Validation, Writing – review and editing.

## Footnotes

1
https://www.randomizer.org
