# Supplementary material for: Influence of specific collagen peptides and 12-week concurrent training on recovery-related biomechanical characteristics following exercise-induced muscle damage—A randomized controlled trial
Source: Front Nutr. 2023 Nov 16;10:1266056. doi: 10.3389/fnut.2023.1266056 (PMC10687431; doi:10.3389/fnut.2023.1266056)

MVC

Test time = pre, post, 24h, 48h
Study time = before, after 12 weeks
Group = SCP, PLA

Assume sphericity?	No
Alpha	0,05

Source of Variation	P value	Significant?	Greenhouse Geisser ε
Test time	<0,0001	Yes	0,4074
Group	0,481	No	
Study time	<0,0001	Yes	1
Test time x Group	0,011	Yes	
Test time x Study time	<0,0001	Yes	0,6443
Group x Study time	0,507	No	
Test time x Group x Study time	0,005	Yes	

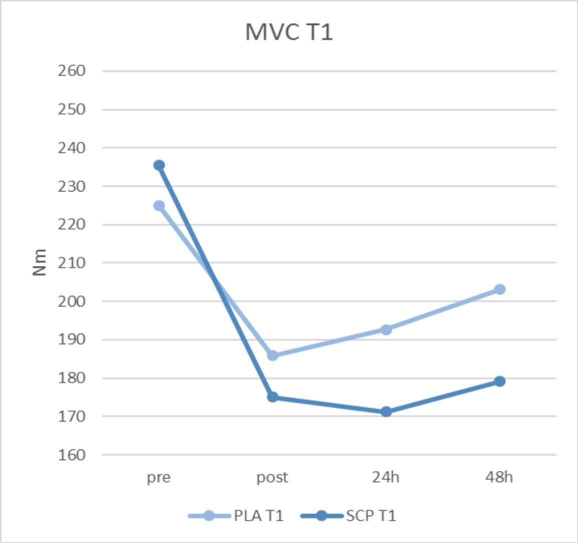

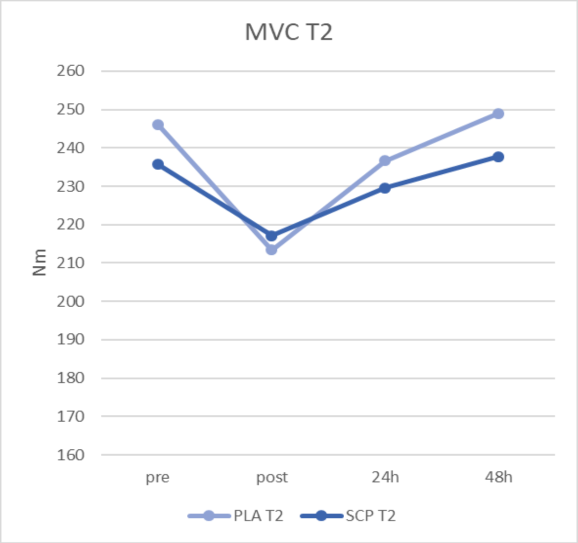

Reduced models:

Test times	Source of Variation	P value	η²p
pre/post	Test time x Group x Study time	0,0014	0,239

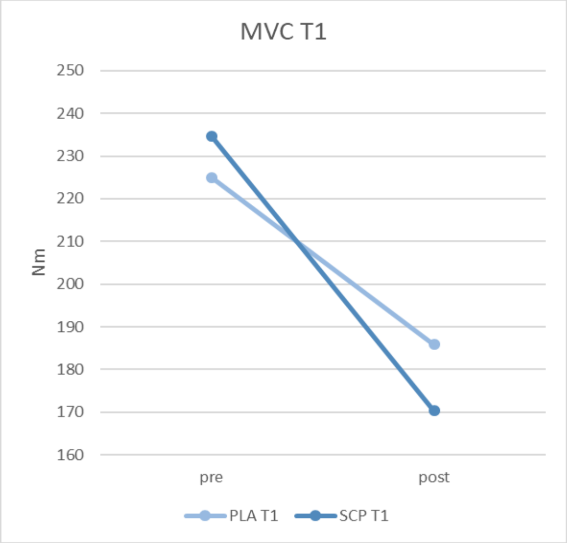

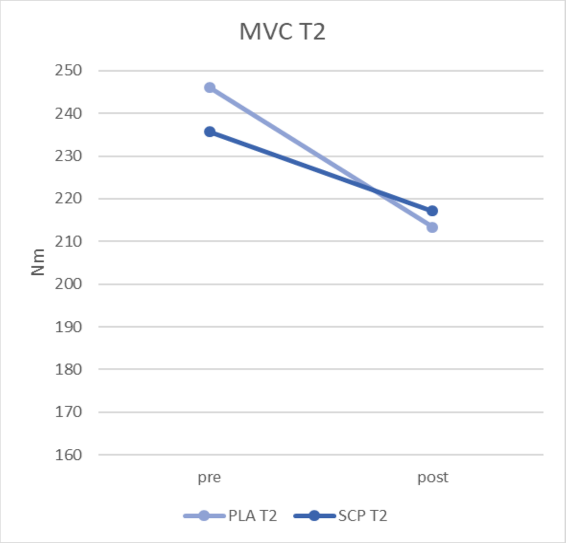

Test times	Source of Variation	P value	η²p
pre/24h	Test time x Group x Study time	0,0262	0,124

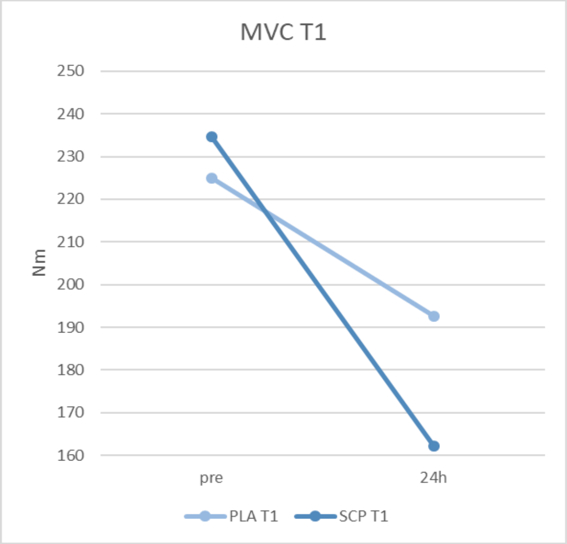

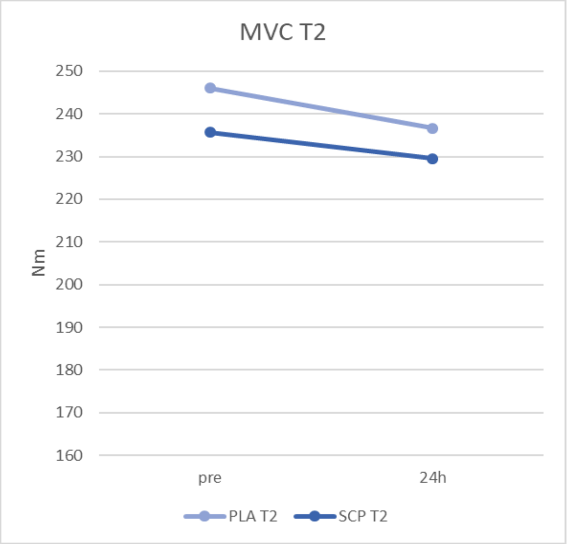

Test times	Source of Variation	P value	η²p
pre/48h	Test time x Group x Study time	0,0259	0,124

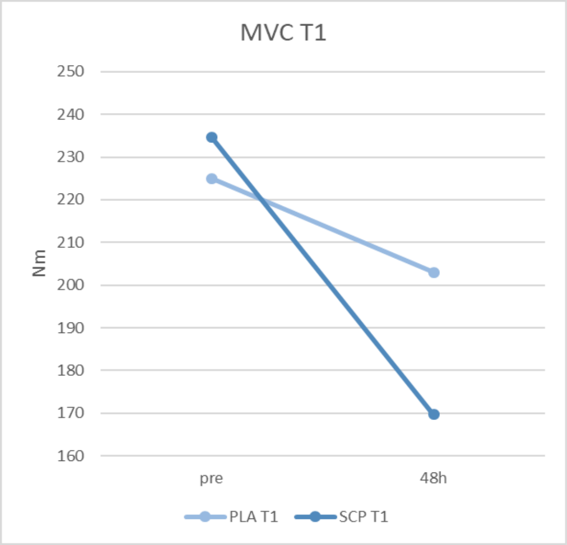

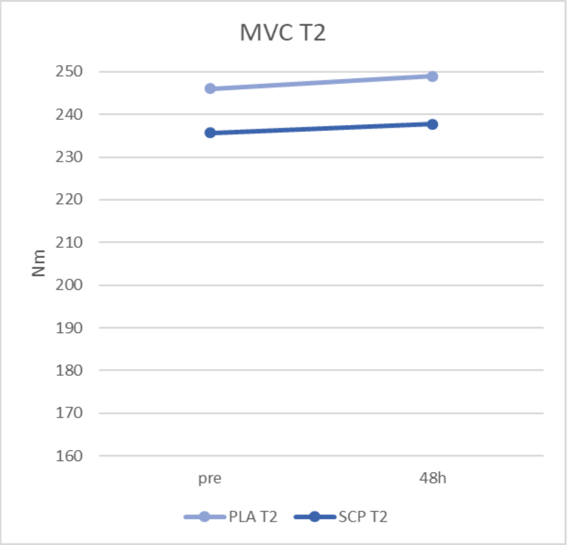

peakRFD 0-100 ms

Test time = pre, post, 24h, 48h
Study time = before, after 12 weeks
Group = SCP, PLA

Assume sphericity?	Yes
Alpha	0,05

Source of Variation	P value	Significant?
Test time	<0,0001	Yes
Group	0,964	No
Study time	0,004	Yes
Test time x Group	0,131	No
Test time x Study time	<0,0001	Yes
Group x Study time	0,0068	Yes
Test time x Group x Study time	0,0004	Yes

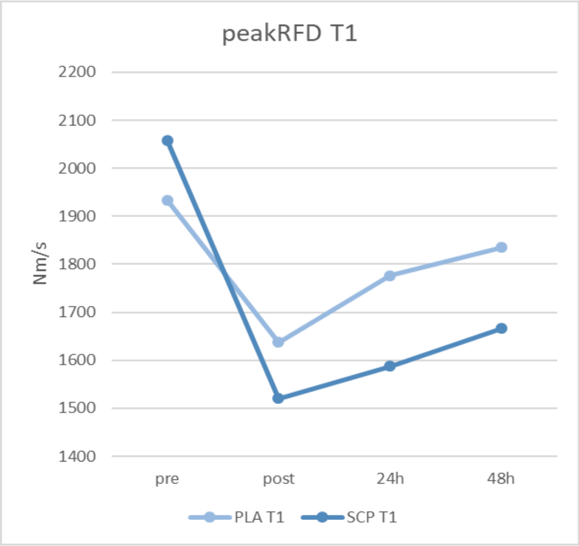

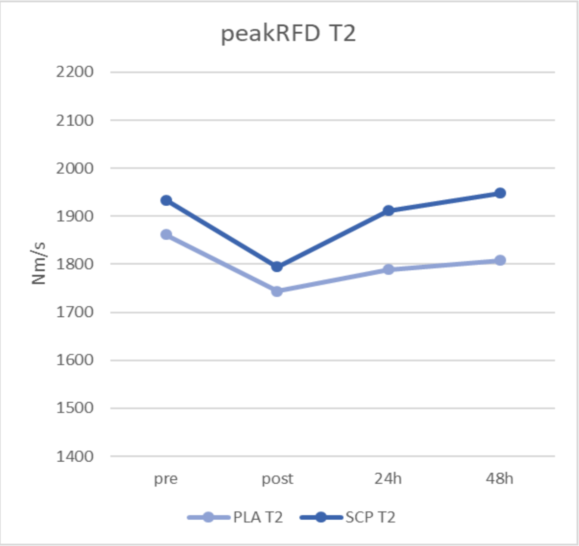

Reduced models:

Test times	Source of Variation	P value	η²p
pre/post	Test time x Group x Study time	0,0084	0,169

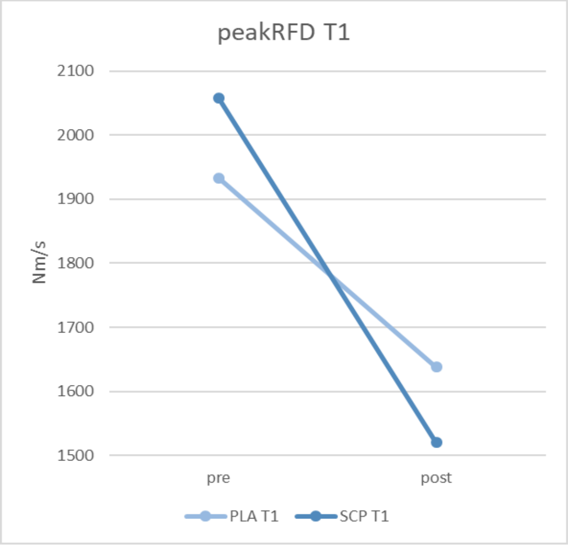

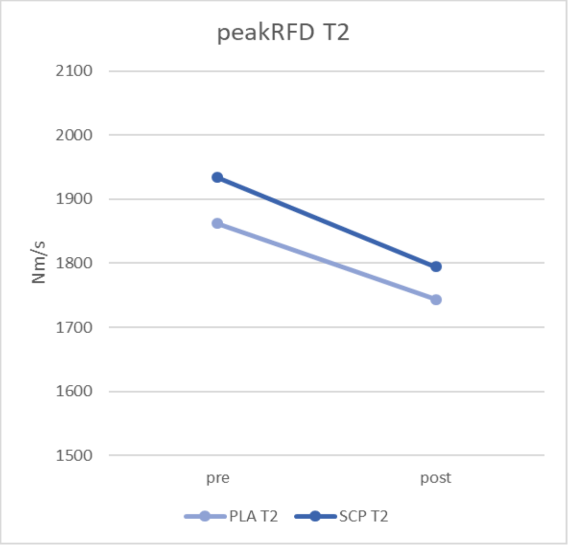

Test times	Source of Variation	P value	η²p
pre/24h	Test time x Group x Study time	0,0019	0,226

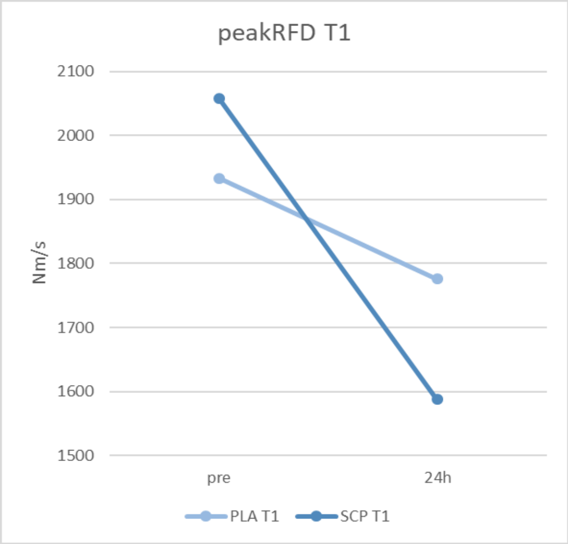

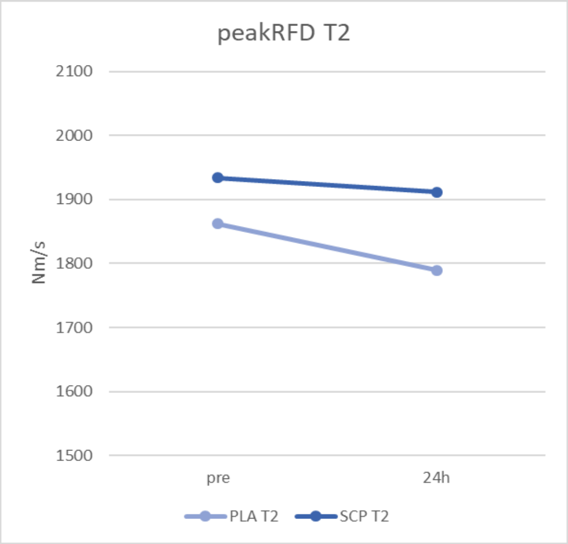

Test times	Source of Variation	P value	η²p
pre/48h	Test time x Group x Study time	0,0016	0,233

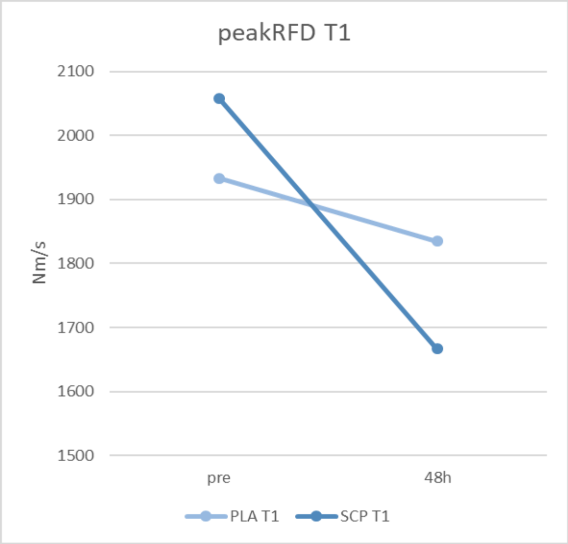

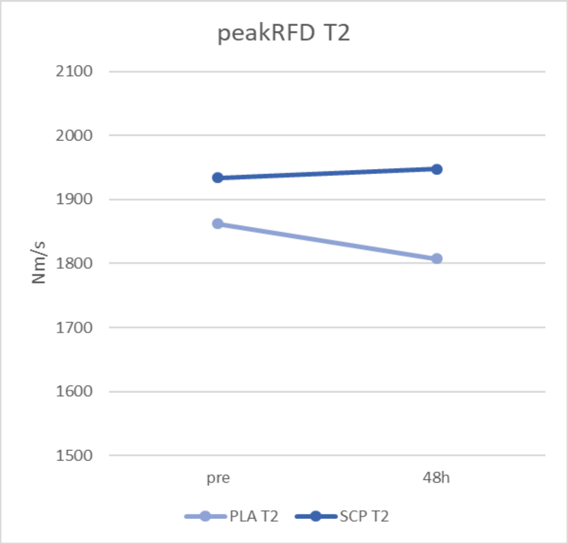

RFD 0-50 ms

Test time = pre, post, 24h, 48h
Study time = before, after 12 weeks
Group = SCP, PLA

Assume sphericity?	Yes
Alpha	0,05

Source of Variation	P value	Significant?
Test time	<0,0001	Yes
Group	0,956	No
Study time	0,0101	Yes
Test time x Group	0,2037	No
Test time x Study time	<0,0001	Yes
Group x Study time	0,004	Yes
Test time x Group x Study time	<0,0001	Yes

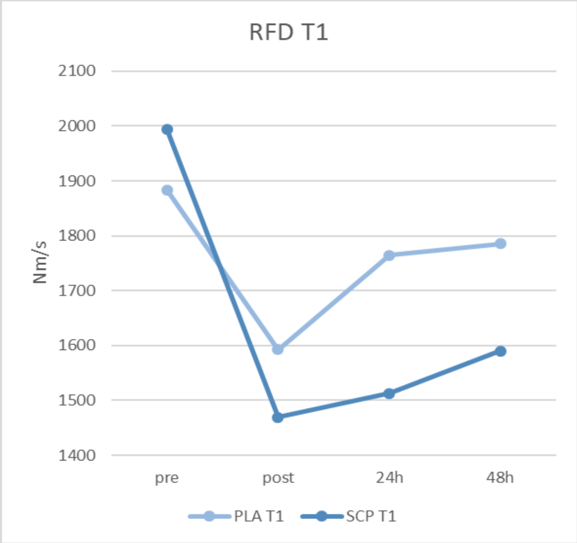

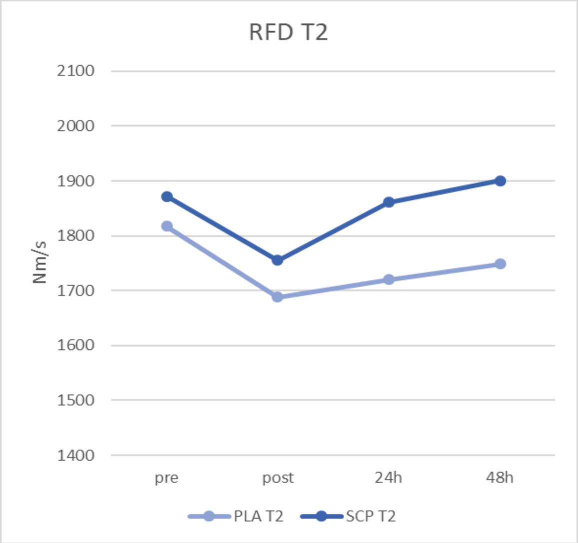

Reduced models:

Test times	Source of Variation	P value	η²p
pre/post	Test time x Group x Study time	0,005	0,191

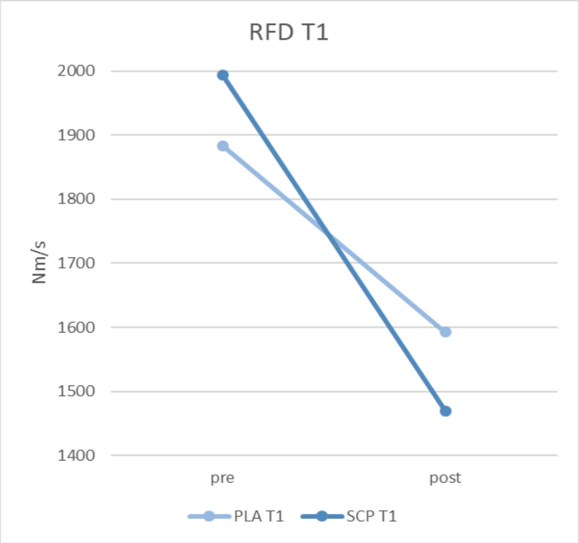

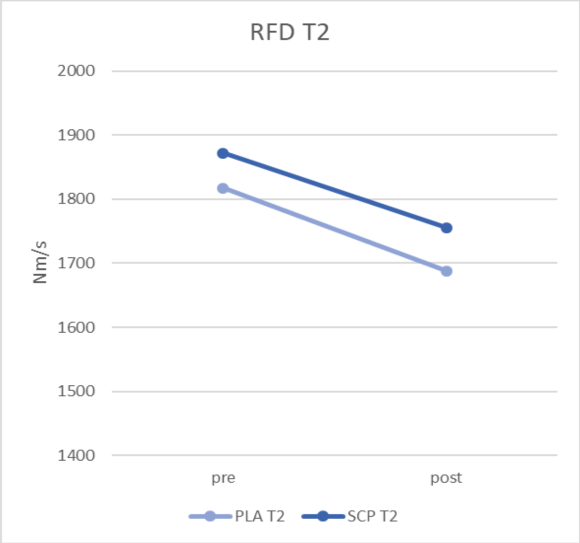

Test times	Source of Variation	P value	$\eta^2 p$
pre/24h	Test time x Group x Study time	0,0005	0,277

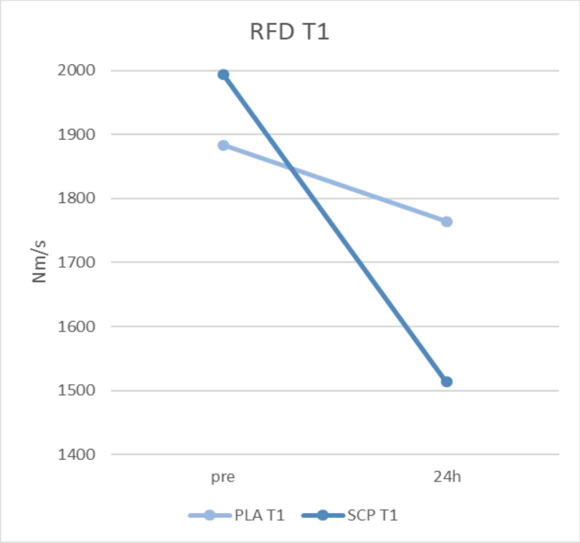

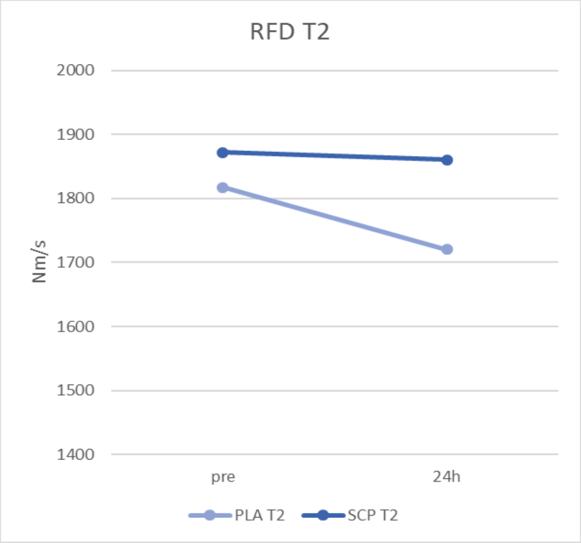

Test times	Source of Variation	P value	$\eta^2 p$
pre/48h	Test time x Group x Study time	0,001	0,251

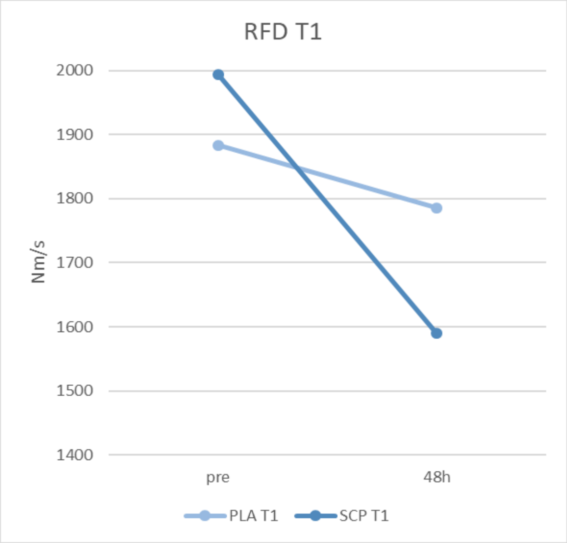

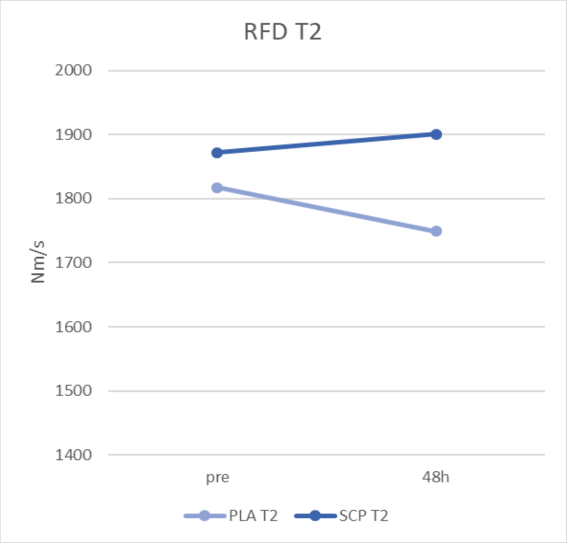

CMJ

Test time = pre, post, 24h, 48h
Study time = before, after 12 weeks
Group = SCP, PLA

Assume sphericity?	No
Alpha	0,05

Source of Variation	P value	Significant?	Greenhouse Geisser ε
Test time	<0,0001	Yes	0,4603
Group	0,4095	No	
Study time	<0,0001	Yes	1
Test time x Group	0,0328	Yes	
Test time x Study time	<0,0001	Yes	0,5747
Group x Study time	0,078	No	
Test time x Group x Study time	0,046	Yes	

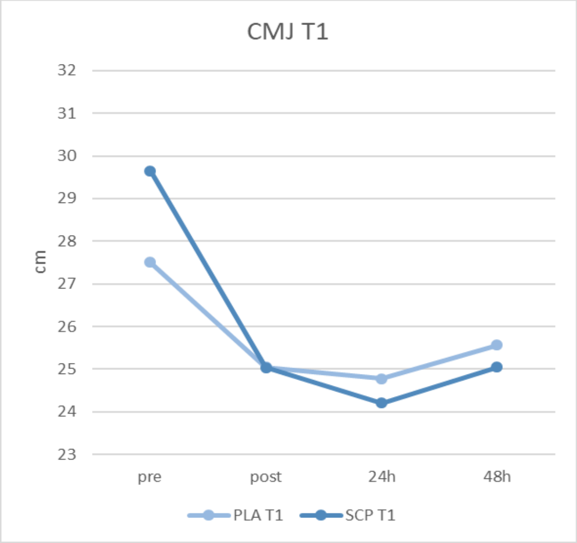

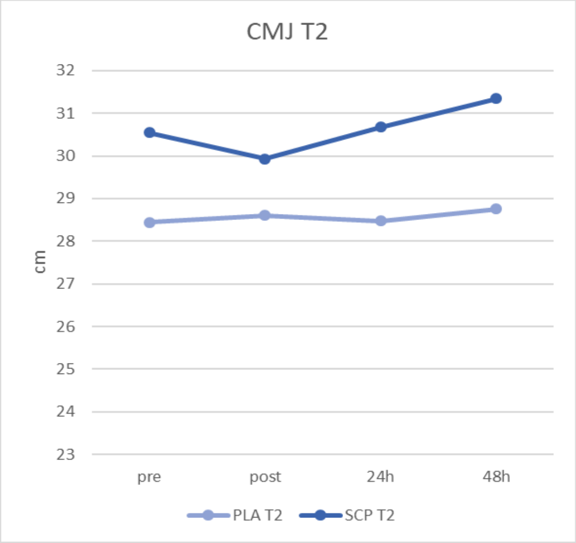

Reduced models:

	Source of Variation	P value	η²p
Test times pre/post	Test time x Group x Study time	0,1174	0,046

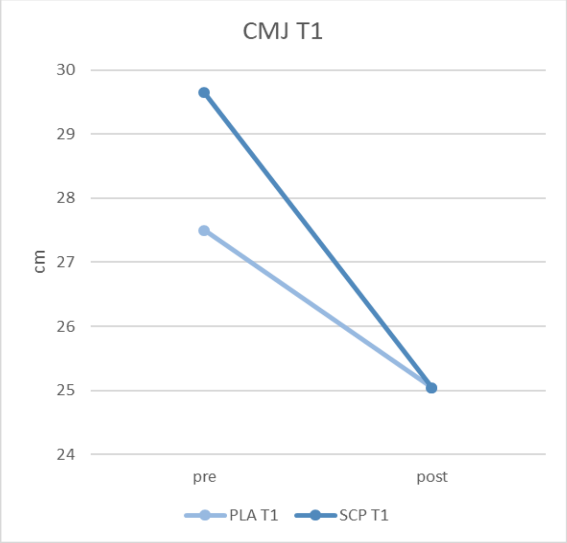

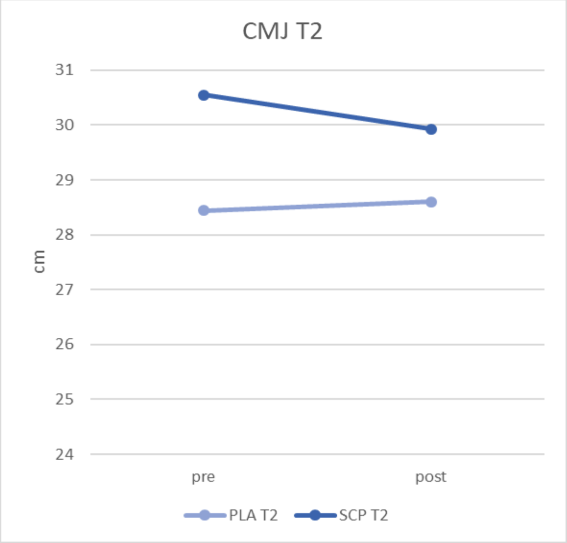

Test times	Source of Variation	P value	η²p
pre/24h	Test time x Group x Study time	0,0729	0,061

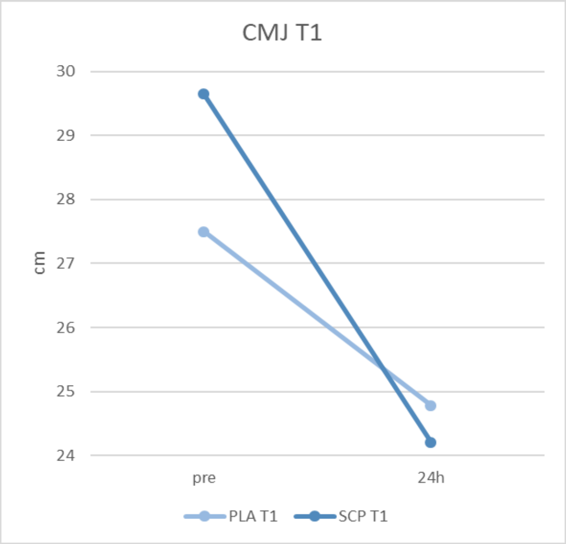

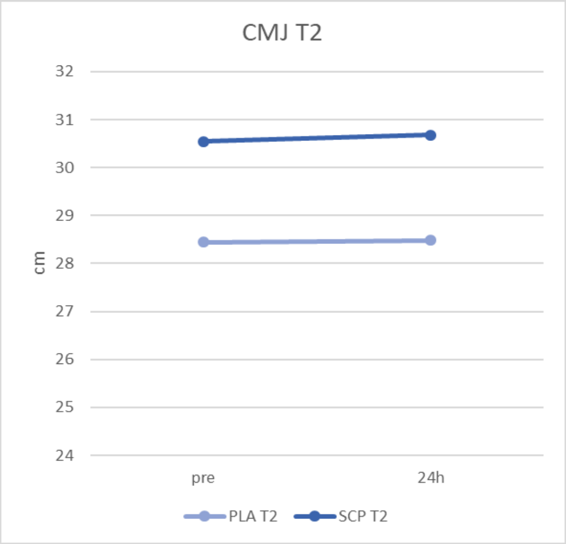

Test times	Source of Variation	P value	η²p
pre/48h	Test time x Group x Study time	0,0224	0,096

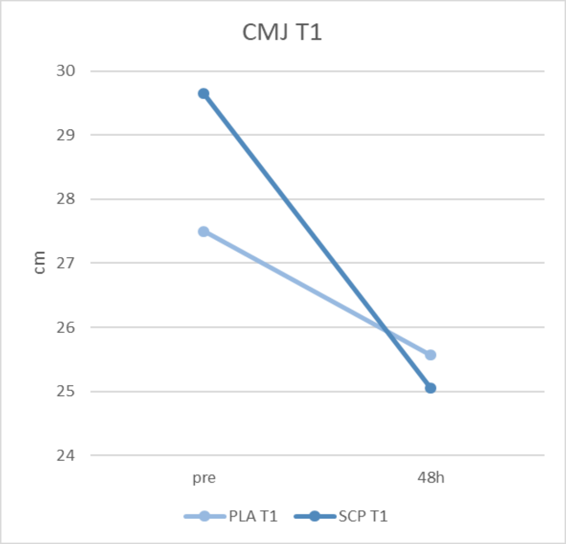

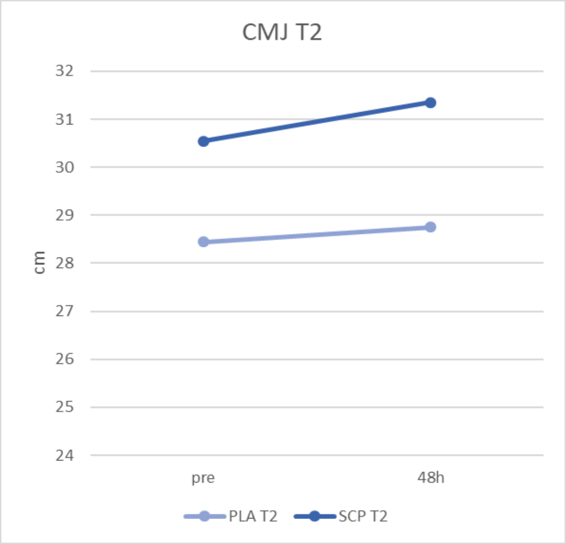

Supplement: Supplementary file 1 [file Data_Sheet_1.pdf]
